# Personalized Infant Risk Prediction for Severe Respiratory Syncytial Virus Lower Respiratory Tract Infection Requiring Intensive Care Unit Admission

**DOI:** 10.1093/ofid/ofae077

**Published:** 2024-02-06

**Authors:** Brittney M Snyder, Niek B Achten, Tebeb Gebretsadik, Pingsheng Wu, Edward F Mitchel, Gabriel Escobar, Louis J Bont, Tina V Hartert

**Affiliations:** Department of Medicine, Vanderbilt University Medical Center, Nashville, Tennessee, USA; Department of Pediatrics, Erasmus University Medical Center, Rotterdam, The Netherlands; Department of Biostatistics, Vanderbilt University Medical Center, Nashville, Tennessee, USA; Department of Medicine, Vanderbilt University Medical Center, Nashville, Tennessee, USA; Department of Biostatistics, Vanderbilt University Medical Center, Nashville, Tennessee, USA; Department of Health Policy, Vanderbilt University Medical Center, Nashville, Tennessee, USA; Division of Research, Kaiser Permanente, Oakland, California, USA; Department of Pediatrics, University Medical Centre Utrecht, Utrecht, The Netherlands; Department of Medicine, Vanderbilt University Medical Center, Nashville, Tennessee, USA; Department of Pediatrics, Vanderbilt University Medical Center, Nashville, Tennessee, USA

**Keywords:** infancy, intensive care unit admission, lower respiratory tract infection, respiratory syncytial virus, risk prediction

## Abstract

**Background:**

Currently, there are no available tools to identify infants at the highest risk of significant morbidity and mortality from respiratory syncytial virus (RSV) lower respiratory tract infection (LRTI) who would benefit most from RSV prevention products. The objective was to develop and internally validate a personalized risk prediction tool for use among all newborns that uses readily available birth/postnatal data to predict RSV LRTI requiring intensive care unit (ICU) admission.

**Methods:**

We conducted a population-based birth cohort study of infants born from 1995 to 2007, insured by the Tennessee Medicaid Program, and who did not receive RSV immunoprophylaxis during the first year of life. The primary outcome was severe RSV LRTI requiring ICU admission during the first year of life. We built a multivariable logistic regression model including demographic and clinical variables available at or shortly after birth to predict the primary outcome.

**Results:**

In a population-based sample of 429 365 infants, 713 (0.2%) had severe RSV LRTI requiring ICU admission. The median age of admission was 66 days (interquartile range, 37–120). Our tool, including 19 variables, demonstrated good predictive accuracy (area under the curve, 0.78; 95% confidence interval, 0.77-0.80) and identified infants who did not qualify for palivizumab, based on American Academy of Pediatrics guidelines, but had higher predicted risk levels than infants who qualified (27% of noneligible infants with >0.16% predicted probabilities [lower quartile for eligible infants]).

**Conclusions:**

We developed a personalized tool that identified infants at increased risk for severe RSV LRTI requiring ICU admission, expected to benefit most from immunoprophylaxis.

## Teaser

Timely identification of infants at highest risk of respiratory syncytial virus–related morbidity is key to prevention. We developed a personalized risk prediction tool using birth/postnatal data that may have applications in promoting immunoprophylaxis to prevent the most severe infant respiratory syncytial virus infections.

Lower respiratory tract infections (LRTIs) in infancy are a major public health issue contributing to thousands of hospitalizations each year in the United States [1]. Immunoprophylaxis with the recently developed, extended half-life, monoclonal antibody nirsevimab can protect both preterm and term infants from respiratory syncytial virus (RSV), the predominant causative pathogen of LRTIs in infancy [2, 3]. Similarly, maternal vaccination against RSV has been shown to effectively prevent severe, medically attended LRTI through at least 90 days of life [4]. The US Centers for Disease Control and Prevention recommends maternal vaccination or infant immunoprophylaxis administration (rarely both) to prevent RSV-associated LRTI among infants [5]. Maternal vaccination is only recommended at 32 to 36 weeks’ gestation during September through January in most of the continental United States. Nirsevimab is recommended for all infants born at <34 weeks’ gestation. Additionally, nirsevimab is recommended for infants aged <8 months born during or entering their first RSV season whose mother was not vaccinated or for which receipt is unknown, or for infants born <14 days after maternal vaccination [5].

Most RSV-associated hospitalizations are among healthy, term infants who have not been eligible for RSV immunoprophylaxis [6–10]. Therefore, there is a clear need for improved means of identifying infants at increased risk for severe RSV LRTIs in countries that recommend RSV prevention products to all infants and in those that limit to high-risk infants. A tool to promptly identify both preterm and term infants who might benefit most from immunoprophylaxis will be particularly beneficial in implementing optimal and cost-effective use, particularly during times of limited availability.

Although several risk prediction tools for RSV LRTI have been developed [11–23], implementation is lacking because of various factors. First, most tools have focused on preterm infants and are, therefore, not generalizable to term infants. Second, tools that use information not routinely collected in clinical care, unavailable at birth, or unavailable at RSV LRTI presentation are impractical for allocating immunoprophylaxis. Third, hospitalization is often used as a broad predictive outcome despite considerable variation in RSV LRTI severity among hospitalized infants. Currently, there are no available tools to identify infants at highest risk of significant morbidity and mortality from RSV LRTI who would yield the greatest benefit from prevention products.

We aimed to develop and internally validate an online, freely available prediction tool including variables routinely available in clinical practice to identify infants at or shortly after birth who are at increased risk for severe RSV LRTI requiring intensive care unit (ICU) admission. Focusing on the most severe RSV LRTI cases allowed for identifying infants in whom the rationale for immunoprophylaxis was strongest and would most likely be cost-effective. To ensure compatibility with nirsevimab and maternal vaccination, our tool was developed for use in all infants, including those traditionally defined as high risk as well as healthy, term infants. We included predictors available at or shortly after birth to permit prompt risk assessment and allocation of immunoprophylaxis.

## METHODS

### Study Population

Our study population included a subset of infants in the Prevention of RSV: Impact on Morbidity and Asthma cohort [24] who were born 1 January 1995 to 31 December 2007, continuously enrolled in the Tennessee Medicaid Program (TennCare), and followed longitudinally through the first year of life. For this analysis, infants who received RSV immunoprophylaxis (Supplementary Table 1) during the first year of life or had a birth hospitalization length of stay of >365 days were excluded.

### Patient Consent Statement

Because this study used existing administrative data that were deidentified and there was no contact with study subjects, study participant written consent was not obtained. The study protocol was approved by the Vanderbilt University Medical Center and Tennessee Department of Health institutional review boards.

### Data Collection

Our primary outcome was severe RSV LRTI requiring ICU admission, defined as acute bronchiolitis or RSV pneumonia ICU hospitalization occurring any time after birth hospitalization within the first year of life. RSV LRTI hospitalization was identified from *International Classification of Diseases, Ninth Revision, Clinical Modification*, diagnosis codes 466.1× (acute bronchiolitis) or 480.1 (pneumonia resulting from RSV) in any diagnostic field for inpatient or other hospital care. We have previously validated this algorithm based on viral identification of RSV [25]. ICU admission was identified for RSV LRTI hospitalizations using health care procedural codes (Supplementary Table 1).

Predictors were selected a priori based on clinical relevance and availability at or near birth from administrative records and birth certificates. For the tool to inform clinical decision making as early in life as possible, we only included predictors that could be captured within the first 30 days of life. Birth weight, gestational age, birth month, infant sex, delivery method, type of birth (singleton, twin, triplet or more), 5-minute Apgar score, number of living siblings, maternal age, maternal education, maternal region of residence (urban, suburban, or rural), and maternal smoking during pregnancy were captured from birth certificates. Continuous positive airway pressure (CPAP) and ventilation during birth hospitalization, as well as comorbidities known to increase the risk for severe RSV LRTIs (Down syndrome, cyanotic heart disease, bronchopulmonary dysplasia, congenital anomalies of the respiratory system, cystic fibrosis, human immunodeficiency virus, and neurologic/neuromuscular disorders), [26] were determined from *International Classification of Diseases, Ninth Revision, Clinical Modification,* and Current Procedural Terminology codes (Supplementary Table 1). Length of birth hospitalization was calculated using date of birth and the infant discharge date. Single imputation methods were used to assign infant discharge date for 62% of infants in whom these data were missing, as previously described [24].

### Statistical Analysis

We used multivariable logistic regression to build a tool for predicting risk of severe RSV LRTI requiring ICU admission in the first year of life. We prespecified the model with 19 demographic and clinical predictors (Supplementary Table 2). To account for nonlinear associations, we used restricted cubic splines to model continuous predictors, as appropriate (Supplementary Figure 1). We reported the effect of each predictor included in the model on risk of severe RSV LRTI requiring ICU admission as an odds ratio adjusted for all other predictors. We determined the relative contribution of each predictor to the final model using χ^2^ values subtracting the individual predictor's respective degrees of freedom.

We assessed the predictive accuracy of the model using discrimination and calibration statistics. We measured model discrimination using area under the receiver operating characteristic curve (AUC) and model calibration using internal model validation with 500 bootstrapped resamples. We plotted the calibration curve using actual versus predicted probability of severe RSV LRTI requiring ICU admission and calculated the calibration intercept and slope. Last, we developed an online tool and nomogram to aid in the translation of our model by allowing health care providers and researchers to easily calculate individual risk estimates for severe RSV LRTI requiring ICU admission in the first year of life.

We carried out 2 sensitivity analyses to evaluate the performance of our model using the AUC metric: (1) among a subset of high-risk infants whose gestational age was <37 weeks and (2) including maternal asthma, an important risk factor for RSV LRTI in infancy [27] as a covariate in the model. The second sensitivity analysis was performed among a subset of infants whose mothers were also enrolled in TennCare from 180 days before the last menstrual period to date of delivery and had maternal asthma ascertained.

Analyses were conducted using R statistical software, version 4.3.2 (R Foundation for Statistical Computing, Vienna, Austria). Results are reported in compliance with the Transparent Reporting of a multivariable prediction model for Individual Prognosis or Diagnosis criteria (Supplementary Material) [28]. Additional details on study methodology can be found in the Supplementary Material.

## RESULTS

Of the 429 365 infants included in our study population (Supplementary Figure 2), 713 (0.2%) had severe RSV LRTI requiring ICU admission during the first year of life. The median age at ICU admission for RSV LRTI was 66 days (interquartile range, 37-120 days). Infants with severe RSV LRTI requiring ICU admission were more likely to be male; born in the later months of the year; and have mothers who smoked during pregnancy, had less education, and lived in an urban environment compared with infants without severe RSV LRTI requiring ICU admission (Table 1). Infants with severe RSV LRTI requiring ICU admission were also more likely to have lower birth weights, be delivered via cesarean section, plural birth (ie, non-singleton), lower gestational age at delivery, ventilated or on CPAP during birth hospitalization, and have bronchopulmonary dysplasia or cyanotic heart disease.

**Table 1.** Characteristics of the Study Population of Infants Born 1995–2007 With Continuous Enrollment in Tennessee Medicaid and who did not Receive RSV Immunoprophylaxis During the First Year of Life Stratified by RSV LRTI Requiring Intensive Care Unit Admission Status

	Infants Without Severe RSV LRTI	Infants With Severe RSV LRTI	Total
Sample size, n (%)	428 652 (100)	713 (0)	429 365 (100)
Demographic predictors			
Infant sex, n (%)			
Male	219 181 (51)	420 (59)	219 601 (51)
Female	209 466 (49)	293 (41)	209 759 (49)
Missing, n (%)	5 (0)	0 (0)	5 (0)
Birth month, median (IQR)	7 (4–10)	9 (3–11)	7 (4–10)
Missing, n (%)	0 (0)	0 (0)	0 (0)
Number of living siblings at delivery, median (IQR)	1 (0–2)	1 (0–2)	1 (0–2)
Missing, n (%)	1058 (0)	0 (0)	1058 (0)
Maternal smoking during pregnancy, n (%)	114 449 (27)	277 (39)	114 726 (27)
Missing, n (%)	681 (0)	1 (0)	682 (0)
Maternal age at delivery (y), median (IQR)	22 (20–26)	22 (20–26)	22 (20–26)
Missing, n (%)	570 (0)	1 (0)	571 (0)
Maternal education at delivery, n (%)			
No high school diploma	157 502 (37)	342 (48)	157 844 (37)
High school diploma	185 457 (43)	283 (40)	185 740 (43)
At least some college	84 511 (20)	86 (12)	84 597 (20)
Missing, n (%)	1182 (0)	2 (0)	1184 (0)
Maternal region of residence at delivery, n (%)			
Urban	148 316 (35)	284 (40)	148 600 (35)
Suburban	110 661 (26)	192 (27)	110 853 (26)
Rural	168 995 (39)	237 (33)	169 232 (39)
Missing, n (%)	680 (0)	0 (0)	680 (0)
Clinical predictors			
Birth weight (g), median (IQR)	3232 (2892–3572)	2919 (2353–3374)	3232 (2892–3572)
Missing, n (%)	0 (0)	0 (0)	0 (0)
Delivery method, n (%)			
Vaginal	322 936 (75)	494 (69)	323 430 (75)
Cesarean section	105 588 (25)	219 (31)	105 807 (25)
Missing, n (%)	128 (0)	0 (0)	128 (0)
Type of birth, n (%)			
Singleton	419 910 (98)	669 (94)	420 579 (98)
Twin	8614 (2)	42 (6)	8656 (2)
Triplet or more	125 (0)	–	–
Missing, n (%)	3 (0)	–	–
Gestational age (wk), median (IQR)	39 (38–40)	38 (36–39)	39 (38–40)
Missing, n (%)	0 (0)	0 (0)	0 (0)
Apgar score, median (IQR)	9 (9–9)	9 (9–9)	9 (9–9)
Missing, n (%)	3059 (1)	7 (1)	3066 (1)
CPAP during birth hospitalization, n (%)	1321 (0)	15 (2)	1336 (0)
Missing, n (%)	0 (0)	0 (0)	0 (0)
Ventilation during birth hospitalization, n (%)	2762 (1)	45 (6)	2807 (1)
Missing, n (%)	0 (0)	0 (0)	0 (0)
Bronchopulmonary dysplasia, n (%)	719 (0)	17 (2)	736 (0)
Missing, n (%)	0 (0)	0 (0)	0 (0)
Congenital anomalies of the respiratory system, n (%)	613 (0)	–	–
Missing, n (%)	0 (0)	0 (0)	0 (0)
Cyanotic heart disease, n (%)	569 (0)	13 (2)	582 (0)
Missing, n (%)	0 (0)	0 (0)	0 (0)
Down syndrome, n (%)	348 (0)	–	–
Missing, n (%)	0 (0)	0 (0)	0 (0)

­­­­­–, Data suppressed (n < 10). Length of birth hospitalization is not listed as single imputation was used to assign 62% of infants for which infant discharge date was missing.

Abbreviations: CPAP, continuous positive airway pressure; IQR, interquartile range; LRTI, lower respiratory tract infection; RSV, respiratory syncytial virus.

**Figure 1.**
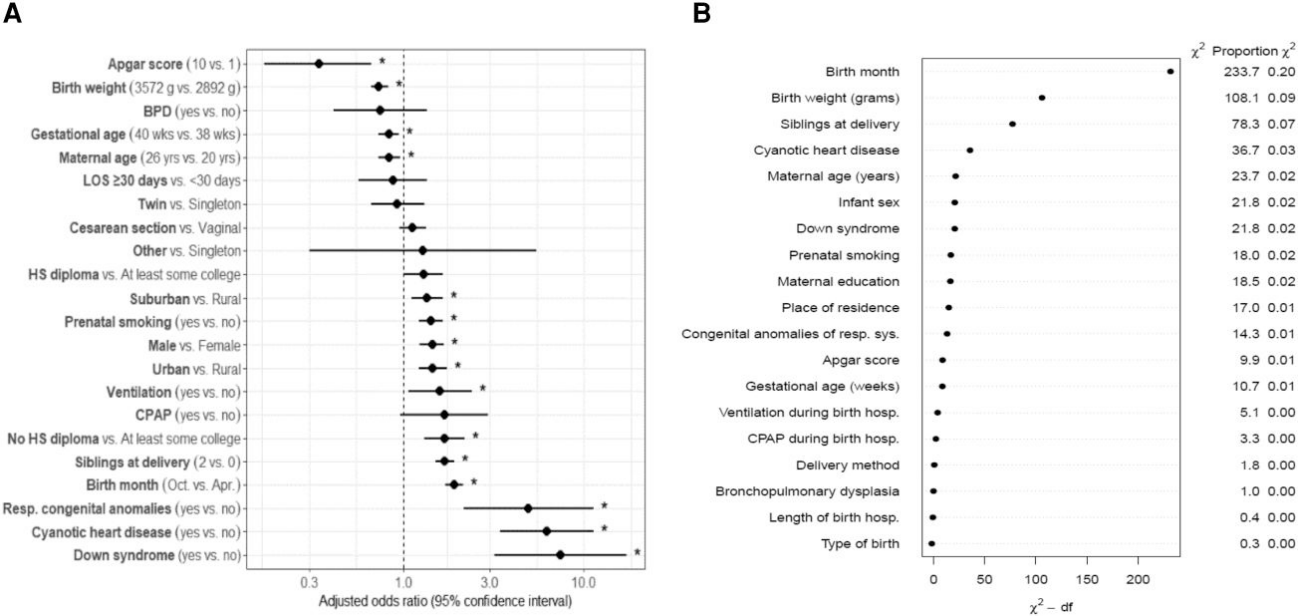
*A*, Association between predictors and risk of severe respiratory syncytial virus (RSV) lower respiratory tract infection (LRTI) requiring intensive care unit (ICU) admission in the first year of life in the study population (n = 429 365). *B*, Relative importance of each predictor included in the model on risk of severe RSV LRTI requiring ICU admission in the first year of life. BPD, bronchopulmonary dysplasia; CPAP, continuous positive airway pressure during birth hospitalization; hosp, hospitalization; HS, high school; LOS, birth hospitalization length of stay; resp, respiratory; resp sys, respiratory system. *A*, Adjusted odds ratios were estimated using multivariable logistic regression adjusting for all other model predictors. The model included infant sex, birth month, number of living siblings at delivery, maternal smoking during pregnancy, maternal age at delivery, maternal education at delivery, maternal region of residence at delivery, birth weight, delivery method, type of birth, gestational age, Apgar score, continuous positive airway pressure during birth hospitalization, ventilation during birth hospitalization, birth hospitalization length of stay, bronchopulmonary dysplasia, congenital anomalies of the respiratory system, cyanotic heart disease, and Down syndrome. *B*, Dots represent chi-square values subtracting the individual predictor's respective degrees of freedom.

Male sex, later birth month, increased number of living siblings at delivery, maternal smoking during pregnancy, mother not having a high school diploma, urban and suburban maternal region of residence at delivery, ventilation during birth hospitalization, congenital anomalies of the respiratory system, cyanotic heart disease, and Down syndrome were associated with statistically significant increased relative odds of severe RSV LRTI requiring ICU admission during the first year of life after adjustment (Figure 1*A* and Supplementary Table 2). Increased maternal age at delivery, higher birth weight, increased gestational age at delivery, and higher 5-minute Apgar score were associated with statistically significant decreased relative odds. Birth month, birth weight, and number of living siblings at delivery ranked highest in relative importance in predicting severe RSV LRTI requiring ICU admission in the first year of life (Figure 1*B*).

**Figure 2.**
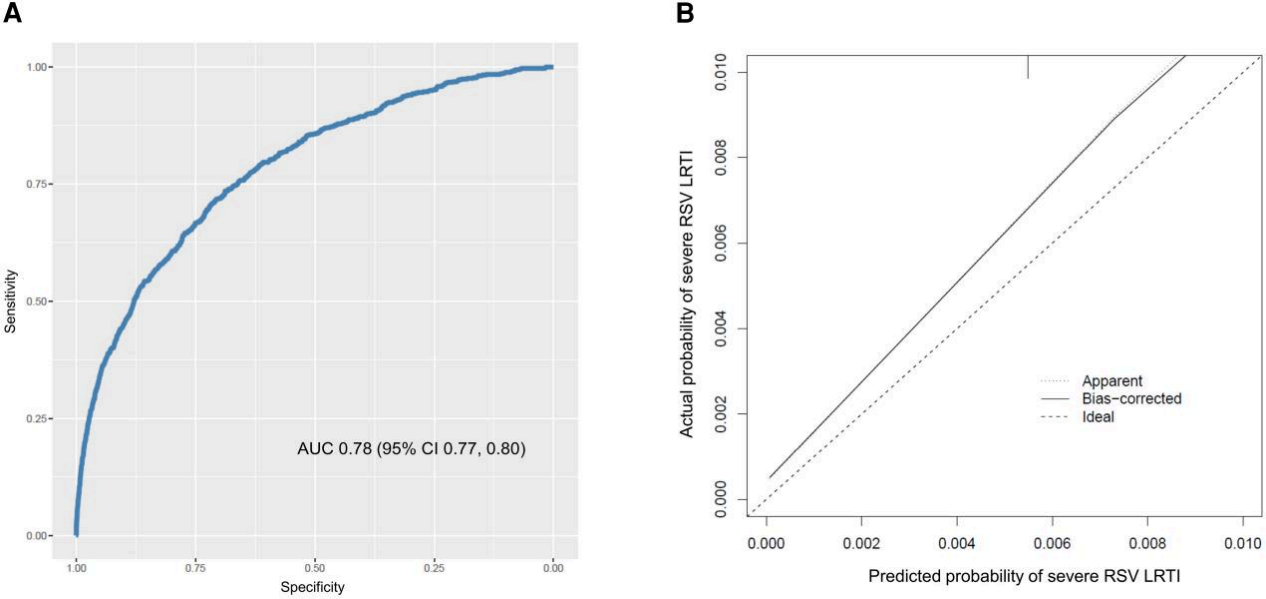
Receiver operating characteristic curve (*A*) and calibration plot (*B*) of our tool for predicting severe respiratory syncytial virus lower respiratory tract infection in the first year of life (n = 429 365).

Supplementary Box 1 outlines our tool's formula for calculating individual predicted risk of severe RSV LRTI requiring ICU admission. Our tool demonstrated good discriminative performance with an AUC of 0.78 (95% confidence interval [CI], 0.77-0.80) (Figure 2*A*). The calibration plot for the internal validation indicated good fit (slope: 0.97), with slight overestimation of the predicted risk of severe RSV LRTI requiring ICU admission in infancy (intercept: −0.17) (Figure 2*B*).

We assessed the discrimination ability of our model (AUC) in 2 sensitivity analyses. Among high-risk, preterm infants (<37 weeks’ gestation, n = 49 209), our tool had an AUC of 0.80 (95% CI, 0.77-0.83). We additionally included maternal history of asthma in a sensitivity analysis including infants whose mothers were also enrolled in TennCare and had maternal asthma ascertained (n = 197 101). Maternal asthma was not associated with severe RSV LRTI in the first year of life (adjusted odds ratio, 1.07; 95% CI, 0.65-1.75), and inclusion of this variable did not improve model performance (AUC, 0.77; 95% CI, 0.75-0.80).

**Figure 3.**
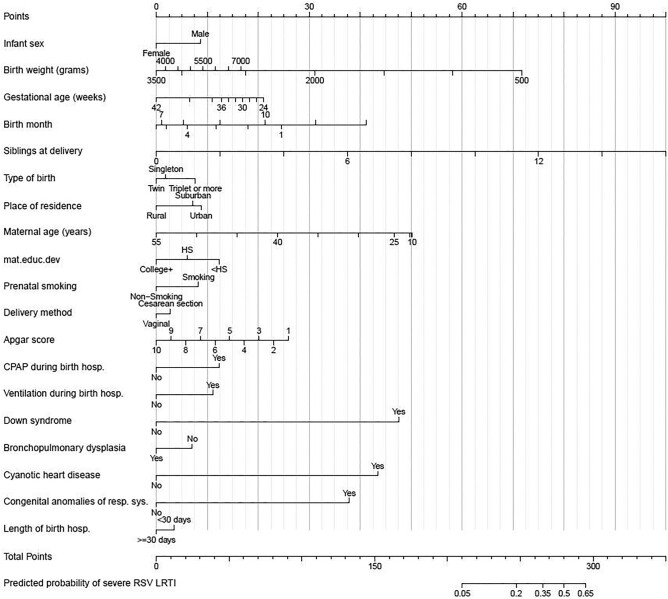
Nomogram for predicted probability of severe respiratory syncytial virus (RSV) lower respiratory tract infection (LRTI) requiring intensive care unit admission in the first year of life. CPAP, continuous positive airway pressure during birth hospitalization; hosp, hospitalization; HS, high school; resp sys, respiratory system. Individuals are assigned points according to the risk factors they possess. Points can then be summed across all predictors to determine the individual's predicted probability of severe RSV LRTI in the first year of life.

To aid in the translation of our tool by allowing health care providers and researchers to easily calculate individual risk estimates for severe RSV LRTI requiring ICU admission in the first year of life, we created a nomogram (Figure 3), online tool (https://cqs.app.vumc.org/shiny/InfantSevereRSVPredictor/), and QR code (Supplementary Figure 3). The predicted probability of severe RSV LRTI requiring ICU admission decreased with increasing gestational age at delivery (Supplementary Figure 4). Similarly, the median predicted probability was higher among infants who met American Academy of Pediatrics palivizumab (RSV immunoprophylaxis—short-acting monoclonal antibody) eligibility during the study period based on birth before 29 weeks’ gestation [8] compared with infants who were not eligible based on having gestational ages ≥29 weeks (0.6% vs 0.1%, respectively; Supplementary Figure 5). However, among these noneligible infants, 27% had predicted probabilities >0.16% (lower quartile for eligible infants). This is further demonstrated in 4 scenarios (Table 2). In scenario A, the predicted risk of severe RSV LRTI requiring ICU admission for an infant who met palivizumab eligibility based on birth before 29 weeks’ gestation was 0.28% (95% CI, 0.16-0.47). We observed higher predicted risk estimates in scenarios B (preterm infant born >29 weeks’ gestation, 1.6%; 95% CI, 1.2-2.3), C (twin birth, 1.1%; 95% CI, 0.74-1.6), and D (term, normal birth weight infant, 0.35%; 95% CI, 0.26-0.49) among infants who were not eligible for palivizumab. If we consider a risk level of >0.28% (observed in scenario A) as high risk, 52 345 infants (12% of our study population) would be identified as high risk by our tool, whereas 355 of 713 (50%) infants with severe RSV LRTI requiring ICU admission in our study population would be identified as high risk. Assuming 80% effectiveness of nirsevimab in preventing RSV LRTI requiring ICU admission [29], this scenario would render a number needed to treat [30] of 185 for this severe outcome alone.

**Table 2.** Scenarios Demonstrating use of our Tool for Calculating Predicted Risk of Severe RSV LRTI Requiring ICU Admission in Infancy by American Academy of Pediatrics Palivizumab Eligibility Guidelines

	Scenarios			
	A. Palivizumab eligible (<29 wk gestation)^a^	B. Not palivizumab eligible (preterm, > 29 wk gestation)	C. Not palivizumab eligible (twin birth)	D. Not palivizumab eligible (term, normal birth weight)
Infant sex	Female	Male	Male	Female
Birth month	June	December	January	January
Number of living siblings at delivery	0	3	2	2
Maternal smoking during pregnancy	No	No	No	Yes
Maternal age at delivery (y)	23	25	20	19
Maternal education at delivery	At least some college	High school diploma	No high school diploma	High school diploma
Maternal region of residence at delivery	Rural	Suburban	Urban	Urban
Birth weight (g)	1000	2150	2250	2900
Gestational age (wk)	28	33	35	38
Delivery method	Vaginal	Vaginal	Cesarean section	Cesarean section
Type of birth	Singleton	Singleton	Twin	Singleton
Apgar score	9	10	8	9
CPAP during birth hospitalization	No	No	No	No
Ventilation during birth hospitalization	No	No	No	No
Birth hospitalization length of stay	<30 d	<30 d	<30 d	<30 d
Bronchopulmonary dysplasia	No	No	No	No
Congenital anomalies of the respiratory system	No	No	No	No
Cyanotic heart disease	No	No	No	No
Down syndrome	No	No	No	No
Predicted risk of severe RSV LRTI requiring ICU admission in infancy	0.28% (95% CI, 0.16-0.47)	1.6% (95% CI, 1.2-2.3)	1.1% (95% CI, 0.74-1.6)	0.35% (95% CI, 0.26-0.49)

Abbreviations: CI, confidence interval; CPAP, continuous positive airway pressure; ICU, intensive care unit; LRTI, lower respiratory tract infection; RSV, respiratory syncytial virus.

^a^Palivizumab eligibility based on American Academy of Pediatrics guidelines (*Pediatrics*, 2014).

## DISCUSSION

Infants requiring ICU care for RSV LRTI represent those who are at highest risk of severe morbidity and death. Following best practices, we developed and internally validated an online, freely available tool to identify infants at increased risk for severe RSV LRTI requiring ICU admission among all infants, those traditionally defined as high risk as well as healthy, term infants. Our tool showed good predictive performance and identified infants who did not qualify for palivizumab under current American Academy of Pediatrics guidelines but had higher predicted risk of severe RSV LRTI requiring ICU admission than infants who were eligible to receive palivizumab based on birth before 29 weeks’ gestation. This tool may have applications in promoting or allocating expensive and/or limited immunoprophylaxis to prevent RSV LRTI requiring ICU admission among infants in the general population or influence vaccine-hesitant families with high-risk infants.

Infant RSV infection is clinically and socioeconomically burdensome, contributing to 50 000 hospitalizations annually in the United States with an average cost of $4000 to $13 000 per hospitalization [7, 31]. Palivizumab was previously the only RSV prophylactic agent available in the United States [32], and it is only recommended for select high-risk infants (eg, a small subset of preterm infants, children with chronic lung disease or congenital heart disease, children with immunodeficiencies) [26]. However, most RSV-associated hospitalizations are among term infants without underlying comorbidities [6, 7]. As of late 2023, both long-acting RSV immunoprophylaxis with nirsevimab and maternal RSV vaccine to protect the infant are available in the United States for use in the general population [33]. Policy decisions regarding use and uptake may vary across the world depending on cost effectiveness and resource allocation, among other factors [34]. Balancing the cost and population health impact of these prevention products is important [23], and there is a clear need for tools to identify infants who are at high risk for severe RSV outcomes for whom these products may have the most benefit. This is especially important in times of limited availability, which was the case for the 2023 through 2024 RSV season in the United States [35]. Our tool addresses this need. Further cost-effectiveness analyses, decision curve analyses, and validation in external populations are needed to implement this tool and determine optimal use.

Previously developed tools for prediction of RSV LRTI risk have suffered from a lack of implementation. However, these tools have primarily focused on predicting risk of RSV LRTI in either preterm or term infants selectively [11, 15, 17–19, 21], reducing their generalizability. Our tool was developed for use in the general population, including term and preterm infants, to ensure wide scope and potential impact. Other tools that include predictors that are not routinely captured in clinical care or not available near birth are not practical in making clinical decisions [20], whereas our tool uses information routinely captured and electronically available at or shortly after birth. Tools have also been developed to predict escalation of care at the time of LRTI presentation, usually in an emergency department setting [12, 22]. Although these may be helpful for clinical decision making, they are not useful from a prevention standpoint. To simplify health care application and aid in decision making, we created an online tool that health care systems and providers could use to predict an infant's risk of severe RSV LRTI at birth or during well-child visits in the first few weeks of life, allowing for prompt implementation of immunoprophylaxis. Additionally, researchers could use this tool to identify infants at risk of severe RSV LRTI requiring ICU admission for secondary analyses of randomized controlled trials and real-world effectiveness studies.

Our study has many strengths; however, there are some important limitations. Although use of a large, population-based cohort allowed us to better estimate risk of a rare outcome, there may have been misclassification because of our reliance on administrative codes for outcome and predictor ascertainment and our truncation of predictors to the first 30 days of life. The incidence of severe RSV LRTI requiring ICU admission in our cohort was similar to previous studies [36, 37], which suggests that misclassification of our primary outcome may be minimal. Additionally, because we defined our primary outcome to include the most severe RSV-related hospitalizations, infants who may be misclassified as high risk for RSV LRTI requiring ICU admission are likely still at high risk for severe RSV-related hospitalization (without ICU admission), and, thus, might benefit from preventive interventions. Although we were unable to confirm RSV infection using laboratory results, we used an algorithm to identify RSV LRTI that we have previously validated based on viral identification of RSV [25].

We did not include race and ethnicity in our tool because these variables have been shown to be poor surrogates of social constructs [38]. The mechanism of health disparities is complex, and although differences in infant LRTI risk by race and ethnicity have been previously observed [39], we could not rule out the possibility of exacerbating health inequities by including these variables in our tool. Future studies could address inequities by considering more socially robust and informative social construct variables, such as neighborhood deprivation index and social vulnerability index [40, 41], for inclusion in this tool by using actual infant address rather than region of residence.

We excluded infants who received RSV immunoprophylaxis in the first year of life because their risk profile would differ from the general population. Because of updated American Academy of Pediatrics guidelines on RSV immunoprophylaxis [8], some of the infants who qualified for palivizumab during the period of this study may not qualify under present guidelines. This may have led to potential selection bias through exclusion of a group of high-risk infants. We have previously shown that infants who were eligible and received RSV immunoprophylaxis were at higher risk for RSV LRTI than infants who were eligible and did not receive RSV immunoprophylaxis [42, 43]. Therefore, these excluded infants would likely be identified as high risk for severe RSV LRTI requiring ICU admission by our tool.

Although we aimed to develop a risk prediction tool that was generalizable to infants traditionally defined as high risk as well as healthy infants, our resulting tool may not be generalizable to all populations because of biologic, socioeconomic, and environmental differences [19]. Our use of a Medicaid population may also impact the generalizability of our findings. Additionally, the dataset from which our tool was derived was curated from data collected from 1995 through 2007 births. Changes in risk factors for severe RSV LRTI, such as improved neonatal care leading to better health outcomes for preterm infants, and thresholds for ICU admission may have changed over time. Although health care patterns may have changed, infants admitted to the ICU would at the very least have been hospitalized, and we believe represent those with the most severe RSV disease. External validation, including assessment of how our tool performs when applied to other populations, including more current populations, and additional severe outcomes of RSV LRTI (eg, mechanical ventilation, death), as well as cost-effectiveness analyses, are needed before clinical and research implementation.

Our tool performed well when assessed among high-risk, preterm infants. However, quantifying the predictive ability of a model on the same data from which the model was developed may overpromise its performance because of overfitting [28]. We did not have an external subpopulation of premature infants with characteristics and outcomes similar to our study population to validate the discrimination and gauge our tool's performance in a premature subset without the potential of overfitting. The future validation of our tool's performance in an external population of premature infants is necessary.

The importance of this risk prediction tool crosses multiple domains. First, it addresses the clinical domain in predicting individual risk of severe RSV infection requiring ICU admission. Second, it has public health implications in predicting the number of infants at risk of this outcome in any given year. Last, it has epidemiologic and research implications in predicting RSV infection requiring ICU admission for research and clinical trials.

## Conclusions

The current emphasis of the National Institutes of Health on Precision Medicine and Patient-Centered Outcomes Research Initiatives is that individuals' needs can and should be predicted and met in a personalized, dynamic manner. However, the success of such mandates obviously depends on the development and performance of risk prediction models. Here, we developed and internally validated an online prediction tool to estimate the risk of severe RSV LRTI requiring ICU admission in the first year of life using a large, population-based cohort. Our tool aimed to identify infants traditionally defined as high risk as well as healthy, term infants at or shortly after birth who were at increased risk for severe RSV LRTI requiring ICU admission. RSV-associated hospitalizations requiring ICU-level care results in significant morbidity and identifies infants at highest risk of death. In a rapidly changing era of RSV prevention, this risk prediction tool is a first step in identifying infants in the general population who might benefit most from RSV immunoprophylaxis.

## Supplementary Material

ofae077_Supplementary_Data

## Supplementary Data

Supplementary materials are available at *Open Forum Infectious Diseases* online. Consisting of data provided by the authors to benefit the reader, the posted materials are not copyedited and are the sole responsibility of the authors, so questions or comments should be addressed to the corresponding author.

## Notes

***Acknowledgments.*** We are indebted to the Division of TennCare in the Tennessee Department of Finance and Administration for providing the data.

***Data availability statement.*** Data not publicly available.

## References

[1] Centers for Disease Control. RSV surveillance & research. Available at: https://www.cdc.gov/rsv/research/index.html

[2] Griffin MP, Yuan Y, Takas T, et al Single-dose nirsevimab for prevention of RSV in preterm infants. N Engl J Med 2020; 383:415–25. 32726528 10.1056/NEJMoa1913556

[3] Muller WJ, Madhi SA, Seoane Nuñez B, et al Nirsevimab for prevention of RSV in term and late-preterm infants. N Engl J Med 2023; 388(16):1533–4. 37018470 10.1056/NEJMc2214773

[4] Kampmann B, Madhi SA, Munjal I, et al Bivalent prefusion F vaccine in pregnancy to prevent RSV illness in infants. N Engl J Med 2023; 388(16):1451–64. 37018474 10.1056/NEJMoa2216480

[5] Fleming-Dutra KE, Jones JM, Roper LE, et al Use of the Pfizer respiratory syncytial virus vaccine during pregnancy for the prevention of respiratory syncytial virus-associated lower respiratory tract disease in infants: recommendations of the Advisory Committee on Immunization Practices—United States, 2023. MMWR Morb Mortal Wkly Rep 2023; 72:1115–22. 37824423 10.15585/mmwr.mm7241e1 PMC10578951

[6] Hall CB, Weinberg GA, Iwane MK, et al The burden of respiratory syncytial virus infection in young children. N Engl J Med 2009; 360:588–98. 19196675 10.1056/NEJMoa0804877 PMC4829966

[7] Rha B, Curns AT, Lively JY, et al Respiratory syncytial virus–associated hospitalizations among young children: 2015–2016. Pediatrics 2020; 146:e20193611. 10.1542/peds.2019-3611 PMC12874392 32546583

[8] Updated guidance for palivizumab prophylaxis among infants and young children at increased risk of hospitalization for respiratory syncytial virus infection. Pediatrics 2014; 134: 415–20. 25070315 10.1542/peds.2014-1665

[9] O'Brien KL, Chandran A, Weatherholtz R, et al Efficacy of motavizumab for the prevention of respiratory syncytial virus disease in healthy Native American infants: a phase 3 randomised double-blind placebo-controlled trial. Lancet Infect Dis 2015; 15:1398–408. 26511956 10.1016/S1473-3099(15)00247-9

[10] Caserta MT, O’Leary ST, Munoz FM, Ralston SL. Palivizumab prophylaxis in infants and young children at increased risk of hospitalization for respiratory syncytial virus infection. Pediatrics 2023; 152:e2023061803. 10.1542/peds.2023-061803 37357729

[11] Houben ML, Bont L, Wilbrink B, et al Clinical prediction rule for RSV bronchiolitis in healthy newborns: prognostic birth cohort study. Pediatrics 2011; 127:35–41. 21187309 10.1542/peds.2010-0581

[12] Luo G, Nkoy FL, Gesteland PH, Glasgow TS, Stone BL. A systematic review of predictive modeling for bronchiolitis. Int J Med Inform 2014; 83:691–714. 25106933 10.1016/j.ijmedinf.2014.07.005

[13] Manuel B, Hackbusch M, Tabatabai J, et al RSVpredict: an online tool to calculate the likelihood of respiratory syncytial virus infection in children hospitalized with acute respiratory disease. Pediatr Infect Dis J 2019; 38:678–81. 30724836 10.1097/INF.0000000000002283

[14] Tso CF, Lam C, Calvert J, Mao Q. Machine learning early prediction of respiratory syncytial virus in pediatric hospitalized patients. Front Pediatr 2022; 10:886212. 35989982 10.3389/fped.2022.886212 PMC9385995

[15] Simões EAF, Carbonell-Estrany X, Fullarton JR, et al A predictive model for respiratory syncytial virus (RSV) hospitalisation of premature infants born at 33–35 weeks of gestational age, based on data from the Spanish FLIP study. Respir Res 2008; 9:78. 19063742 10.1186/1465-9921-9-78 PMC2636782

[16] Gebremedhin AT, Hogan AB, Blyth CC, Glass K, Moore HC. Developing a prediction model to estimate the true burden of respiratory syncytial virus (RSV) in hospitalised children in Western Australia. Sci Rep 2022; 12:332. 35013434 10.1038/s41598-021-04080-3 PMC8748465

[17] Sampalis JS, Langley J, Carbonell-Estrany X, et al Development and validation of a risk scoring tool to predict respiratory syncytial virus hospitalization in premature infants born at 33 through 35 completed weeks of gestation. Med Decis Making 2008; 28:471–80. 18556643 10.1177/0272989X08315238

[18] Blanken MO, Paes B, Anderson EJ, et al Risk scoring tool to predict respiratory syncytial virus hospitalisation in premature infants. Pediatr Pulmonol 2018; 53:605–12. 29405612 10.1002/ppul.23960 PMC6099524

[19] Straňák Z, Saliba E, Kosma P, et al Predictors of RSV LRTI hospitalization in infants born at 33 to 35 weeks gestational age: a large multinational study (PONI). PLoS One 2016; 11:e0157446. 27310438 10.1371/journal.pone.0157446 PMC4910988

[20] Rietveld E, Vergouwe Y, Steyerberg EW, et al Hospitalization for respiratory syncytial virus infection in young children: development of a clinical prediction rule. Pediatr Infect Dis J 2006; 25:201–7. 16511380 10.1097/01.inf.0000202135.24485.f8

[21] Paes B, Cole M, Latchman A, Pinelli J. Predictive value of the respiratory syncytial virus risk-scoring tool in the term infant in Canada. Curr Med Res Opin 2009; 25:2191–6. 19604126 10.1185/03007990903126908

[22] Yan J, Zhao L, Zhang T, et al Development and validation of a nomogram for predicting severe respiratory syncytial virus-associated bronchiolitis. BMC Infect Dis 2023; 23:249. 37072700 10.1186/s12879-023-08179-y PMC10114343

[23] Vartiainen P, Jukarainen S, Rhedin SA, et al Risk factors for severe respiratory syncytial virus infection during the first year of life: development and validation of a clinical prediction model. Lancet Digit Health 2023; 5:e821–30. 37890904 10.1016/S2589-7500(23)00175-9

[24] Escobar GJ, Gebretsadik T, Carroll K, et al Adherence to immunoprophylaxis regimens for respiratory syncytial virus infection in insured and Medicaid populations. J Pediatric Infect Dis Soc 2013; 2:205–14. 24921044 10.1093/jpids/pit007 PMC4043196

[25] Turi KN, Wu P, Escobar GJ, et al Prevalence of infant bronchiolitis-coded healthcare encounters attributable to RSV. Health Sci Rep 2018; 1:e91-e. 30623050 10.1002/hsr2.91 PMC6295609

[26] UpToDate. Respiratory syncytial virus infection: prevention in infants and children. Available at: https://www.uptodate.com/contents/respiratory-syncytial-virus-infection-prevention#H104280778

[27] Carroll KN, Gebretsadik T, Griffin MR, et al Maternal asthma and maternal smoking are associated with increased risk of bronchiolitis during infancy. Pediatrics 2007; 119:1104–12. 17545377 10.1542/peds.2006-2837

[28] Moons KG, Altman DG, Reitsma JB, et al Transparent Reporting of a multivariable prediction model for Individual Prognosis or Diagnosis (TRIPOD): explanation and elaboration. Ann Intern Med 2015; 162:W1–W73. 25560730 10.7326/M14-0698

[29] Simões EAF, Madhi SA, Muller WJ, et al Efficacy of nirsevimab against respiratory syncytial virus lower respiratory tract infections in preterm and term infants, and pharmacokinetic extrapolation to infants with congenital heart disease and chronic lung disease: a pooled analysis of randomised controlled trials. Lancet Child Adolesc Health 2023; 7:180–9. 36634694 10.1016/S2352-4642(22)00321-2 PMC9940918

[30] Laupacis A, Sackett DL, Roberts RS. An assessment of clinically useful measures of the consequences of treatment. N Engl J Med 1988; 318:1728–33. 3374545 10.1056/NEJM198806303182605

[31] Smart KA, Lanctôt KL, Paes BA. The cost effectiveness of palivizumab: a systematic review of the evidence. J Med Econ 2010; 13:453–63. 20653398 10.3111/13696998.2010.499749

[32] American Family Physician. AAP updates guidelines on immunoprophylaxis for RSV infection. Available at: https://www.aafp.org/pubs/afp/issues/2010/0901/p542.html

[33] Centers for Disease Control and Prevention. RSV prevention. Available at: https://www.cdc.gov/rsv/about/prevention.html

[34] Karron RA . RSV illness in the young and the old—the beginning of the end? N Engl J Med 2023; 388:1522–4. 37018472 10.1056/NEJMe2302646

[35] Centers for Disease Control and Prevention. Limited availability of nirsevimab in the United States—interim CDC recommendations to protect infants from respiratory syncytial virus (RSV) during the 2023–2024 respiratory virus season. Available at: https://emergency.cdc.gov/han/2023/han00499.asp

[36] McLaurin KK, Farr AM, Wade SW, Diakun DR, Stewart DL. Respiratory syncytial virus hospitalization outcomes and costs of full-term and preterm infants. J Perinatol 2016; 36:990–6. 27490190 10.1038/jp.2016.113 PMC5090170

[37] Ghazaly M, Nadel S. Characteristics of children admitted to intensive care with acute bronchiolitis. Eur J Pediatr 2018; 177:913–20. 29654399 10.1007/s00431-018-3138-6 PMC5958152

[38] Ioannidis JPA, Powe NR, Yancy C. Recalibrating the use of race in medical research. JAMA 2021; 325:623–4. 33492329 10.1001/jama.2021.0003

[39] Inagaki K, Blackshear C, Burns PA, Hobbs CV. Racial/ethnic disparities in the incidences of bronchiolitis requiring hospitalization. Clin Infect Dis 2020; 72:668–74. 10.1093/cid/ciaa113 32020165

[40] Andrews MR, Tamura K, Claudel SE, et al Geospatial analysis of neighborhood deprivation Index (NDI) for the United States by county. J Maps 2020; 16:101–12. 32855653 10.1080/17445647.2020.1750066 PMC7447192

[41] Agency for Toxic Substances and Disease Registry (CDC/ATSDR). CDC/ARSDR SVI fact sheet. Available at: https://www.atsdr.cdc.gov/placeandhealth/svi/fact_sheet/fact_sheet.html

[42] Carroll KN, Gebretsadik T, Escobar GJ, et al Respiratory syncytial virus immunoprophylaxis in high-risk infants and development of childhood asthma. J Allergy Clin Immunol 2017; 139:66–71.e3. 27212083 10.1016/j.jaci.2016.01.055 PMC5074917

[43] Wu P, Escobar GJ, Gebretsadik T, et al Effectiveness of respiratory syncytial virus immunoprophylaxis in reducing bronchiolitis hospitalizations among high-risk infants. Am J Epidemiol 2018; 187:1490–500. 29351636 10.1093/aje/kwy008 PMC6030843

