# Supplementary material for: Personalized Infant Risk Prediction for Severe Respiratory Syncytial Virus Lower Respiratory Tract Infection Requiring Intensive Care Unit Admission
Source: Open Forum Infect Dis. 2024 Feb 6;11(3):ofae077. doi: 10.1093/ofid/ofae077 (PMC10932939; doi:10.1093/ofid/ofae077)
Supplement: ofae077_Supplementary_Data [file ofae077_supplementary_data.docx]

**Personalized infant risk prediction for severe respiratory syncytial virus lower respiratory tract infection requiring intensive care unit admission**

Brittney M. Snyder, Niek B. Achten, Tebeb Gebretsadik, Pingsheng Wu, Edward F. Mitchel, Gabriel Escobar, Louis J. Bont, Tina V. Hartert

**Supplementary Material**

[Methods 2](#_Toc157067191)

[Study Population 2](#_Toc157067192)

[Data Collection 3](#_Toc157067193)

[Statistical Analysis 4](#_Toc157067194)

[References 7](#_Toc157067195)

[Supplementary Table 1 10](#_Toc157067196)

[Supplementary Table 2 12](#_Toc157067197)

[Supplementary Figure 1. 14](#_Toc157067198)

[TRIPOD Checklist 15](#_Toc157067199)

[Supplementary Figure 2 18](#_Toc157067200)

[Supplementary Box 1 19](#_Toc157067201)

[Supplementary Figure 3 20](#_Toc157067202)

[Supplementary Figure 4 21](#_Toc157067203)

[Supplementary Figure 5 22](#_Toc157067204)

# Methods

## ***Study Population***

Our study population included a subset of infants in the Prevention of RSV: Impact on Morbidity and Asthma (PRIMA) cohort who were born January 1, 1995 to December 31, 2007, continuously enrolled in the Tennessee Medicaid Program (TennCare), and followed longitudinally through the first year of life. Continuous enrollment was defined as no more than 90 days of non-enrollment from birth to 365 days of life for the infant. This cohort has been described previously.[1] For this analysis, infants who received RSV immunoprophylaxis during the first year of life were excluded as these infants were at decreased risk of severe respiratory syncytial virus (RSV) lower respiratory tract infection (LRTI) requiring intensive care unit (ICU) admission. RSV immunoprophylaxis was identified from outpatient pharmacy claims and healthcare encounters and was based on prescription fill of RSV intravenous immune globulin (RespiGam) or palivizumab (Synagis) or health care procedural codes (**Supplementary Table 1**). Infants who were eligible for but did not receive RSV immunoprophylaxis were included. Additionally, the study years included time prior to immunoprophylaxis availability and early years during which there was low uptake. As severe RSV LRTI requiring ICU admission must have occurred after birth hospitalization within the first year of life, we further restricted the study population to include infants who had a birth hospitalization length of stay ≤365 days. The study protocol was approved by the Vanderbilt University Medical Center and Tennessee Department of Health Institutional Review Boards.

## ***Data Collection***

For this study, we utilized data from healthcare encounters and linked birth and death certificates. Our primary outcome was severe RSV LRTI, defined as acute bronchiolitis or RSV pneumonia hospitalization requiring ICU admission occurring any time after birth hospitalization within the first year of life. RSV LRTI hospitalization was identified from International Classification of Diseases, Ninth Revision, Clinical Modification*(*ICD-9) diagnosis codes 466.1x (acute bronchiolitis) or 480.1 (pneumonia due to RSV) in any diagnostic field for inpatient or other hospital care. We have previously validated this algorithm based on viral identification of RSV.[2] ICU admission was identified for RSV LRTI hospitalizations using health care procedural codes (**Supplementary Table 1**).

Predictors were selected *a priori* based on clinical relevance and availability at or near birth in administrative records and birth certificates. For the tool to inform clinical decision making as early in life as possible, we only included predictors that could be captured near birth (i.e., within the first 30 days of life). Birth weight, birth month, infant sex, delivery method, type of birth (singleton, twin, triplet or more), 5-minute Apgar score, number of living siblings at delivery, maternal age at delivery, maternal education at delivery, maternal region of residence at delivery, and maternal smoking during pregnancy were captured from birth certificates. Gestational age was calculated using date of last menstrual period (LMP) for whom this information was available from birth certificates (87%). For women in which LMP was not available (13%), we calculated the LMP from the median gestational age in weeks for infants of the same birth weight, birth year, and race.[3-7] Region of residence (urban, suburban, or rural) was determined based on the county listed and categorized according to the 2010 Census Urban and Rural Classification and Urban Area Criteria.[8] Continuous positive airway pressure (CPAP) and ventilation during birth hospitalization, as well as comorbidities known to increase the risk for severe RSV LRTIs (Down syndrome, cyanotic heart disease, bronchopulmonary dysplasia [BPD], congenital anomalies of the respiratory system, cystic fibrosis [CF], human immunodeficiency virus [HIV], and neurologic/neuromuscular disorders),[9] were determined from ICD-9 and Current Procedural Terminology (CPT) codes (**Supplementary Table 1**). Length of birth hospitalization was calculated using date of birth and the infant discharge date. Single imputation methods were used to assign infant discharge date for 62% of infants in whom these data were missing as previously described.[1] For ease of clinical and research use, we dichotomized this predictor as <30 days or ≥30 days. Predictive accuracy was unchanged when length of stay was included as a continuous predictor.

## ***Statistical Analysis***

We used multivariable logistic regression to build a tool for predicting risk of severe RSV LRTI requiring ICU admission in the first year of life. We pre-specified the model with 19 demographic and clinical predictors (**Supplementary Table 2**). Predictors capturing HIV, CF, and neurologic/neuromuscular disorders status were not included in the model as no infants with severe RSV LRTI requiring ICU admission in our study population had these diagnoses. Variable clustering was used to assess collinearity between predictors and decrease redundancy. None of the predictors were found to be collinear (Spearman’s correlation coefficient <0.7 and >-0.7). To account for non-linear associations, we used restricted cubic splines to model continuous predictors, as appropriate (**Supplementary Figure 1**).

Once single imputation was applied for infant length of stay, the variable with the most missing data was Apgar score at birth (1% missing for total population; **Table 1**). For the main model, we carried out a complete case analysis with 422,199 observations (98% of the original sample size [N=429,365]). Sensitivity analyses that did not include infant birth length of stay variable were performed and results were consistent.

We reported the effect of each predictor included in the model on risk of severe RSV LRTI requiring ICU admission as an odds ratio (OR) adjusted for all other predictors, and we calculated the corresponding 95% confidence intervals (CI) using Wald statistics. For continuous predictors, adjusted ORs (aORs) were based on difference of the interquartile range (IQR, 25^th^-75^th^). P-values less than 0.05 were considered statistically significant. We determined the relative contribution of each predictor to the final model using χ^2^ values subtracting the individual predictor’s respective degrees of freedom.

We assessed the predictive accuracy of the model using discrimination and calibration statistics. We measured model discrimination using area under the receiver operating characteristic curve (AUC) and model calibration (i.e., goodness of fit) using internal model validation with 500 bootstrapped resamples. We plotted the calibration curve using actual versus predicted probability of severe RSV LRTI requiring ICU admission, and we calculated the calibration intercept and slope. A calibration intercept <0 is indicative of model overestimation, an intercept >0 is indicative of model underestimation, and a calibration slope <1 is indicative of model overfitting.[10-12] Lastly, we developed a nomogram and online tool to aid in the translation of our model by allowing healthcare providers and researchers to easily calculate individual risk estimates for severe RSV LRTI requiring ICU admission in the first year of life.

As maternal asthma is also an important risk factor for RSV LRTI in infancy,[13] this variable was included in the prediction model in a sensitivity analysis performed among a subset of infants whose mothers were also enrolled in TennCare from 180 days prior to LMP to date of delivery and had maternal asthma ascertained. Maternal asthma was ascertained using a previously validated algorithm.[3, 7, 14-19] Women with any of the following conditions were considered to have maternal asthma: 1) ≥1 ICD-9-CM diagnosis of 493.xx in any of the diagnosis fields for inpatient, other hospital care, or emergency department visit; 2) ≥2 ICD-9-CM diagnoses of 493.xx in any of the diagnosis fields for outpatient physician visit claims separated by ≥30 days; 3) ≥2 prescription fills for any short-acting beta-agonist in the 180 days prior to the LMP to the date of delivery; 4) ≥2 prescription fills for montelukast in the 180 days prior the LMP to the date of delivery and in a 365-day period prior to the Food and Drug Administration approval of montelukast for allergic rhinitis; 5) ≥1 prescription of any other asthma-specific medication (i.e., inhaled corticosteroid, long-acting beta agonist, combination corticosteroid/long-acting beta agonist, leukotriene modifying agent) in the 180 days prior to the LMP to the date of delivery.

Analyses were conducted using R statistical software, version 4.3.2 (R Foundation for Statistical Computing, Vienna, Austria). Results are reported in compliance with the Transparent Reporting of a multivariable prediction model for Individual Prognosis or Diagnosis (TRIPOD) criteria.[20]

# References

1. Escobar GJ, Gebretsadik T, Carroll K, et al. Adherence to Immunoprophylaxis Regimens for Respiratory Syncytial Virus Infection in Insured and Medicaid Populations. J Pediatric Infect Dis Soc **2013**; 2(3): 205-14.

2. Turi KN, Wu P, Escobar GJ, et al. Prevalence of infant bronchiolitis-coded healthcare encounters attributable to RSV. Health Sci Rep **2018**; 1(12): e91-e.

3. Turi KN, Gebretsadik T, Ding T, et al. Dose, Timing, and Spectrum of Prenatal Antibiotic Exposure and Risk of Childhood Asthma. Clin Infect Dis **2021**; 72(3): 455-62.

4. Hayes RM, Wu P, Shelton RC, et al. Maternal antidepressant use and adverse outcomes: a cohort study of 228,876 pregnancies. Am J Obstet Gynecol **2012**; 207(1): 49.e1-9.

5. Carroll KN, Griffin MR, Gebretsadik T, Shintani A, Mitchel E, Hartert TV. Racial differences in asthma morbidity during pregnancy. Obstet Gynecol **2005**; 106(1): 66-72.

6. Cooper WO, Hernandez-Diaz S, Arbogast PG, et al. Major congenital malformations after first-trimester exposure to ACE inhibitors. N Engl J Med **2006**; 354(23): 2443-51.

7. Snyder BM, Patterson MF, Gebretsadik T, et al. Association between asthma status and prenatal antibiotic prescription fills among women in a Medicaid population. J Asthma **2022**; 59(10): 2100-7.

8. United States Census Bureau. 2010 census urban and rural classification and urban area criteria. Available at: <https://www.census.gov/programs-surveys/geography/guidance/geo-areas/urban-rural/2010-urban-rural.html>. Accessed 22 September 2023.

9. UpToDate. Respiratory syncytial virus infection: Prevention in infants and children. Available at: <https://www.uptodate.com/contents/respiratory-syncytial-virus-infection-prevention#H104280778>. Accessed 22 September 2023.

10. Lamain-de Ruiter M, Kwee A, Naaktgeboren CA, et al. External validation of prognostic models to predict risk of gestational diabetes mellitus in one Dutch cohort: prospective multicentre cohort study. BMJ **2016**; 354: i4338.

11. Steyerberg EW, Vickers AJ, Cook NR, et al. Assessing the performance of prediction models: a framework for traditional and novel measures. Epidemiology **2010**; 21(1): 128-38.

12. Donovan BM, Breheny PJ, Robinson JG, et al. Development and validation of a clinical model for preconception and early pregnancy risk prediction of gestational diabetes mellitus in nulliparous women. PloS one **2019**; 14(4): e0215173-e.

13. Carroll KN, Gebretsadik T, Griffin MR, et al. Maternal asthma and maternal smoking are associated with increased risk of bronchiolitis during infancy. Pediatrics **2007**; 119(6): 1104-12.

14. Wu P, Dupont WD, Griffin MR, et al. Evidence of a causal role of winter virus infection during infancy in early childhood asthma. Am J Respir Crit Care Med **2008**; 178(11): 1123-9.

15. Wakefield DB, Cloutier MM. Modifications to HEDIS and CSTE algorithms improve case recognition of pediatric asthma. Pediatr Pulmonol **2006**; 41(10): 962-71.

16. Hartert TV, Togias A, Mellen BG, Mitchel EF, Snowden MS, Griffin MR. Underutilization of controller and rescue medications among older adults with asthma requiring hospital care. J Am Geriatr Soc **2000**; 48(6): 651-7.

17. Hartert TV, Speroff T, Togias A, et al. Risk factors for recurrent asthma hospital visits and death among a population of indigent older adults with asthma. Ann Allergy Asthma Immunol **2002**; 89(5): 467-73.

18. Talbot TR, Hartert TV, Mitchel E, et al. Asthma as a risk factor for invasive pneumococcal disease. N Engl J Med **2005**; 352(20): 2082-90.

19. Donovan BM, Abreo A, Ding T, et al. Dose, Timing, and Type of Infant Antibiotic Use and the Risk of Childhood Asthma. Clin Infect Dis **2020**; 70(8): 1658-65.

20. Moons KG, Altman DG, Reitsma JB, et al. Transparent Reporting of a multivariable prediction model for Individual Prognosis or Diagnosis (TRIPOD): explanation and elaboration. Ann Intern Med **2015**; 162(1): W1-73.

Supplementary Table 1. *International Classification of Diseases, 9th Revision, Clinical Modification* (ICD-9), Current Procedural Terminology (CPT), and Healthcare Common Procedure Coding System (HCPCS) codes used to identify infants with respiratory syncytial virus (RSV) immunoprophylaxis, intensive care unit (ICU) admission, continuous positive airway pressure and/or mechanical ventilation during birth hospitalization, and/or with comorbidities known to increase the risk for severe RSV lower respiratory tract infections.

| **Disease/Condition** | **ICD-9 Code(s)** | **CPT code(s)** | **HCPCS codes** |
| --- | --- | --- | --- |
| RSV immunoprophylaxis |  | 90378, 90379 | C9003, J1565 |
| ICU admission |  | 99291, 99292 |  |
| Continuous positive airway pressure, mechanical ventilation |  | 93.90, 93.91, 96.7xx |  |
| Comorbidities |  |  |  |
| Bronchopulmonary dysplasia | 770.7 |  |  |
| Congenital anomalies of the respiratory system |  |  |  |
| Web of larynx | 748.2 |  |  |
| Other anomalies of larynx, trachea, and bronchus | 748.3 |  |  |
| Anomalies of diaphragm | 756.6 |  |  |
| Tracheoesophageal fistula, esophageal atresia  and stenosis | 750.3 |  |  |
| Cystic fibrosis | 277.0x |  |  |
| Human immunodeficiency virus | 042 |  |  |
| Neurologic/neuromuscular disorders |  |  |  |
| Hemiplegia and hemiparesis | 342.xx |  |  |
| Infantile cerebral palsy | 343.x |  |  |
| Quadriplegia and quadraparesis | 344.0x |  |  |
| Paraplegia | 344.1 |  |  |
| Diplegia of upper limbs | 344.2 |  |  |
| Monoplegia of lower limb | 344.3x |  |  |
| Monoplegia of upper limb | 344.4x |  |  |
| Unspecified monoplegia | 344.5 |  |  |
| Encephalopathy, not elsewhere classified | 348.3x |  |  |
| Congenital hereditary muscular dystrophy | 359.0 |  |  |
| Hereditary progressive muscular dystrophy | 359.1 |  |  |
| Cyanotic heart disease |  |  |  |
| Common truncus | 745.0 |  |  |
| Tetralogy of fallot | 745.2 |  |  |
| Common ventricle | 745.3 |  |  |
| Anomalies of pulmonary valve congenital | 746.0x |  |  |
| Tricuspid atresia and stenosis, congenital | 746.1 |  |  |
| Ebstein’s anomaly | 746.2 |  |  |
| Hypoplastic left heart syndrome | 746.7 |  |  |
| Congenital anomalies of great veins | 747.4x |  |  |
| Situs inversus | 759.3 |  |  |
| Down syndrome | 758.0 |  |  |

For continuous positive airway pressure and mechanical ventilation, we included any diagnostic field for inpatient or hospitalization. For all other predictors, we included any diagnostic field for inpatient, outpatient, or hospitalization during the first 30 days of life.

Supplementary Table 2. Association between predictors and risk of severe respiratory syncytial virus (RSV) lower respiratory tract infection (LRTI) requiring intensive care unit admission in the first year of life.

| **Predictor** | **OR (95% CI)** | **aOR (95% CI)** |
| --- | --- | --- |
| Infant sex |  |  |
| Male | 1.37 (1.18, 1.59)* | 1.44 (1.23, 1.67)* |
| Female | REF | REF |
| Birth month | 1.88 (1.68-2.10)* | 1.90 (1.70-2.13)* |
| Number of living siblings at delivery | 1.50 (1.37-1.64)* | 1.69 (1.51-1.90)* |
| Maternal smoking during pregnancy | 1.74 (1.50-2.03)* | 1.42 (1.21-1.66)* |
| Maternal age at delivery | 0.96 (0.88-1.04) | 0.83 (0.73-0.96)* |
| Maternal education at delivery |  |  |
| No high school diploma | 2.13 (1.68-2.70)* | 1.68 (1.30-2.18)* |
| High school diploma | 1.50 (1.18, 1.91)* | 1.29 (1.00, 1.66) |
| At least some college | REF | REF |
| Maternal region of residence at delivery |  |  |
| Urban | 1.37 (1.15-1.62)* | 1.45 (1.21-1.73)* |
| Suburban | 1.24 (1.02-1.50)* | 1.35 (1.11-1.65)* |
| Rural | REF | REF |
| Birth weight | 0.66 (0.60-0.73)* | 0.73 (0.66-0.82)* |
| Gestational age | 0.71 (0.63-0.80)* | 0.83 (0.73-0.94)* |
| Delivery method |  |  |
| Vaginal | REF | REF |
| Cesarean section | 1.36 (1.16-1.59)* | 1.12 (0.95-1.33) |
| Type of birth |  |  |
| Singleton | REF | REF |
| Twin | 3.06 (2.24-4.18)* | 0.92 (0.66-1.30) |
| Triplet or more | 10.04 (2.48-40.68)* | 1.28 (0.30-5.43) |
| Apgar score | 0.05 (0.03-0.08)* | 0.34 (0.17-0.66)* |
| CPAP during birth hospitalization | 6.95 (4.16-11.63)* | 1.68 (0.96-2.92) |
| Ventilation during birth hospitalization | 10.39 (7.66-14.08)* | 1.59 (1.06-2.39)* |
| Birth hospitalization length of stay |  |  |
| <30 days | REF | REF |
| ≥30 days | 7.97 (6.19-10.27)* | 0.87 (0.56-1.34) |
| Bronchopulmonary dysplasia | 14.54 (8.94-23.65)* | 0.74 (0.41-1.34) |
| Congenital anomalies of the respiratory system | 5.93 (2.64-13.29)* | 4.91 (2.15-11.21)* |
| Cyanotic heart disease | 13.97 (8.02-24.33)* | 6.22 (3.44-11.22)* |
| Down syndrome | 10.44 (4.64-23.49)* | 7.40 (3.19-17.17)* |

OR, odds ratio; aOR, adjusted odds ratio; CI, confidence interval; REF, reference group; CPAP, continuous positive airway pressure.

Odds ratios and two-sided p-values were estimated using univariate logistic regression. Adjusted odds ratios and two-sided p-values were estimated using multivariable logistic regression. Each variable was adjusted for all other variables within the table. Odds ratios for continuous predictors were based on increases in interquartile range differences (25^th^-75^th^ percentile).

*p<0.05

Supplementary Figure 1. Continuous predictors and log odds of severe respiratory syncytial virus lower respiratory tract infection requiring intensive care unit admission in the first year of life in the study population (n=429,365).


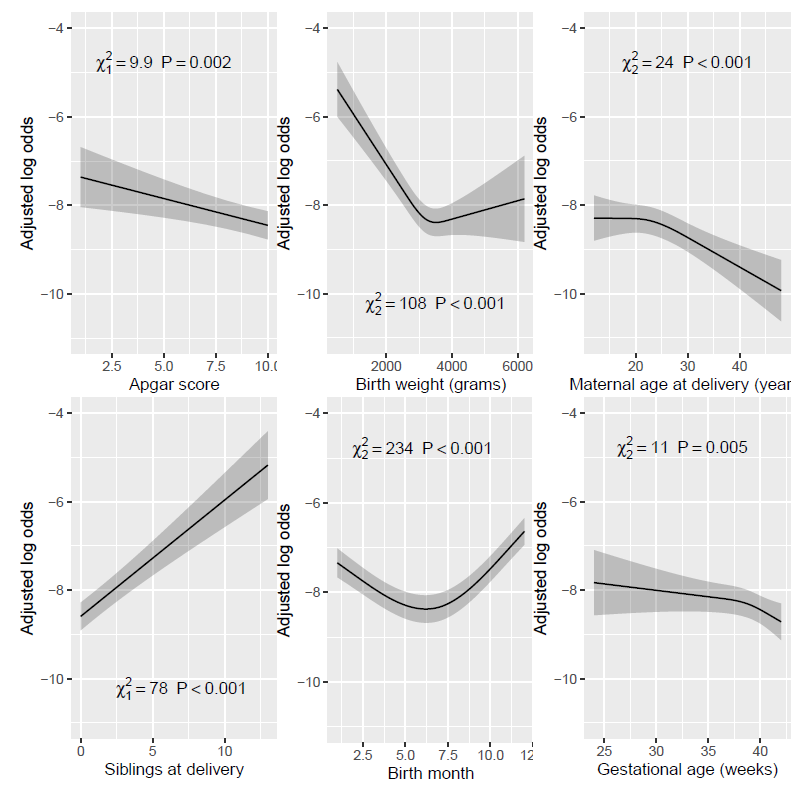
Log odds were calculated using multivariable logistic regression. Each predictor was adjusted for all other model predictors. The model included infant sex, birth month, number of living siblings at delivery, maternal smoking during pregnancy, maternal age at delivery, maternal education at delivery, maternal region of residence at delivery, birth weight, delivery method, type of birth, gestational age, Apgar score, continuous positive airway pressure during birth hospitalization, ventilation during birth hospitalization, birth hospitalization length of stay, bronchopulmonary dysplasia, congenital anomalies of the respiratory system, cyanotic heart disease, and Down syndrome.

TRIPOD Checklist**: Prediction model development and validation.**

| **Section/Topic** | **Item** |  | **Checklist Item** | **Page** |
| --- | --- | --- | --- | --- |
| **Title and abstract** | | | | |
| Title | 1 | D;V | Identify the study as developing and/or validating a multivariable prediction model, the target population, and the outcome to be predicted. | 1 |
| Abstract | 2 | D;V | Provide a summary of objectives, study design, setting, participants, sample size, predictors, outcome, statistical analysis, results, and conclusions. | 3-4 |
| **Introduction** | | | | |
| Background and objectives | 3a | D;V | Explain the medical context (including whether diagnostic or prognostic) and rationale for developing or validating the multivariable prediction model, including references to existing models. | 5-6 |
|  | 3b | D;V | Specify the objectives, including whether the study describes the development or validation of the model or both. | 6 |
| **Methods** | | | | |
| Source of data | 4a | D;V | Describe the study design or source of data (e.g., randomized trial, cohort, or registry data), separately for the development and validation data sets, if applicable. | 6-7 |
|  | 4b | D;V | Specify the key study dates, including start of accrual; end of accrual; and, if applicable, end of follow-up. | 6 |
| Participants | 5a | D;V | Specify key elements of the study setting (e.g., primary care, secondary care, general population) including number and location of centres. | 6 |
|  | 5b | D;V | Describe eligibility criteria for participants. | 6-7; Supp. Material |
|  | 5c | D;V | Give details of treatments received, if relevant. | N/A |
| Outcome | 6a | D;V | Clearly define the outcome that is predicted by the prediction model, including how and when assessed. | 7 |
|  | 6b | D;V | Report any actions to blind assessment of the outcome to be predicted. | N/A |
| Predictors | 7a | D;V | Clearly define all predictors used in developing or validating the multivariable prediction model, including how and when they were measured. | 7-8; Supp. Material |
|  | 7b | D;V | Report any actions to blind assessment of predictors for the outcome and other predictors. | N/A |
| Sample size | 8 | D;V | Explain how the study size was arrived at. | N/A |
| Missing data | 9 | D;V | Describe how missing data were handled (e.g., complete-case analysis, single imputation, multiple imputation) with details of any imputation method. | Supp. Material |
| Statistical analysis methods | 10a | D | Describe how predictors were handled in the analyses. | 8-9; Supp. Material |
|  | 10b | D | Specify type of model, all model-building procedures (including any predictor selection), and method for internal validation. | 8-9; Supp. Material |
|  | 10c | V | For validation, describe how the predictions were calculated. | 8-9; Supp. Material |
|  | 10d | D;V | Specify all measures used to assess model performance and, if relevant, to compare multiple models. | 8-9 |
|  | 10e | V | Describe any model updating (e.g., recalibration) arising from the validation, if done. | N/A |
| Risk groups | 11 | D;V | Provide details on how risk groups were created, if done. | 12 |
| Development vs. validation | 12 | V | For validation, identify any differences from the development data in setting, eligibility criteria, outcome, and predictors. | N/A |
| **Results** | | | | |
| Participants | 13a | D;V | Describe the flow of participants through the study, including the number of participants with and without the outcome and, if applicable, a summary of the follow-up time. A diagram may be helpful. | 10; Supp. Fig. 2 |
|  | 13b | D;V | Describe the characteristics of the participants (basic demographics, clinical features, available predictors), including the number of participants with missing data for predictors and outcome. | Table 1 |
|  | 13c | V | For validation, show a comparison with the development data of the distribution of important variables (demographics, predictors and outcome). | N/A |
| Model development | 14a | D | Specify the number of participants and outcome events in each analysis. | 10 |
|  | 14b | D | If done, report the unadjusted association between each candidate predictor and outcome. | Supp. Table 2 |
| Model specification | 15a | D | Present the full prediction model to allow predictions for individuals (i.e., all regression coefficients, and model intercept or baseline survival at a given time point). | Supp. Box 1 |
|  | 15b | D | Explain how to the use the prediction model. | 11-12, Fig 3, online tool, Table 2 |
| Model performance | 16 | D;V | Report performance measures (with CIs) for the prediction model. | 11 |
| Model-updating | 17 | V | If done, report the results from any model updating (i.e., model specification, model performance). | N/A |
| **Discussion** | | | | |
| Limitations | 18 | D;V | Discuss any limitations of the study (such as nonrepresentative sample, few events per predictor, missing data). | 14-16 |
| Interpretation | 19a | V | For validation, discuss the results with reference to performance in the development data, and any other validation data. | N/A |
|  | 19b | D;V | Give an overall interpretation of the results, considering objectives, limitations, results from similar studies, and other relevant evidence. | 13-14 |
| Implications | 20 | D;V | Discuss the potential clinical use of the model and implications for future research. | 13-17 |
| **Other information** | | | | |
| Supplementary information | 21 | D;V | Provide information about the availability of supplementary resources, such as study protocol, Web calculator, and data sets. | 12 |
| Funding | 22 | D;V | Give the source of funding and the role of the funders for the present study. | 19 |

Reference: Collins GS, *BMJ* 2015. *Items relevant only to the development of a prediction model are denoted by D, items relating solely to a validation of prediction model are denoted by V, and items relating to both are denoted D;V.

Supplementary Figure 2**.** Flow diagram of study population.

Infants born 1995-2007 included in PRIMA cohort

n=458,837

Exclusions

Not continuously enrolled in Tennessee Medicaid Program

n=17,697

RSV immunoprophylaxis receipt during the first year of life

N=11,754

Birth length of stay >365 days

n=21

**Final study population**

**n=429,365**

RSV, respiratory syncytial virus.

Supplementary Box 1. Formula to calculate individual predicted risk of severe respiratory syncytial virus (RSV) lower respiratory tract infection (LRTI) requiring intensive care unit (ICU) admission in the first year of life.

Predicted risk of severe RSV LRTI requiring ICU admission = $\frac{1}{1+exp(-X\beta)}$ where,

X$\hat{\beta}$ =

-1.1 +

-0.36[Infant sex: Female] +

-0.0011[Birth weight] +

5x10^-10^([Birth weight] – 2559)^3^ +

-1x10^-9^([Birth weight] – 3232)^3^ +

5.2x10^-10^([Birth weight] – 3883)^3^ +

-0.029[Gestational age] +

-0.0024([Gestational age] – 36)^3^ +

0.0061([Gestational age] – 39)^3^ +

-0.0036([Gestational age] – 41)^3^ +

-0.27[Birth month] +

0.0051([Birth month] – 2)^3^ +

-0.012([Birth month] – 7)^3^ +

0.0064([Birth month] – 11)^3^ +

0.26[Number of living siblings at delivery] +

0.24[Type of birth: Triplet or more] +

-0.079[Type of birth**:** Twin] +

0.37[Maternal region of residence at delivery: Urban] +

0.3[Maternal region of residence at delivery: Suburban] +

-0.0013[Maternal age at delivery] +

- 0.00042[Maternal age at delivery -18)^3^ +

0.00061[Maternal age at delivery -22]^3^ +

-0.00019[Maternal age at delivery – 31]^3^ +

0.52[Maternal education at delivery: No high school diploma] +

0.26[Maternal education at delivery: High school diploma] +

0.35[Maternal smoking during pregnancy] +

0.12[Delivery method: Cesarean section] +

-0.12[Apgar score] +

0.52[CPAP during birth hospitalization] +

0.47[Ventilation during birth hospitalization] +

2[Down syndrome] +

-0.3[Bronchopulmonary dysplasia] +

1.8[Cyanotic heart disease] +

1.6[Congenital anomalies of the respiratory system] +

-0.14[Birth hospitalization length of stay: ≥30 days]

Supplementary Figure 3. QR code for online tool to calculate individual risk estimates for severe respiratory syncytial virus requiring intensive care unit admission in the first year of life.


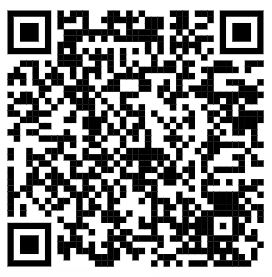


Supplementary Figure 4. Predicted probability of severe respiratory syncytial virus (RSV) lower respiratory tract infection (LRTI) requiring intensive care unit admission in the first year of life by gestational age at delivery.





Predicted probability was estimated using univariate logistic regression. The vertical dashed line indicates 29 weeks gestation (i.e., RSV immunoprophylaxis eligible according to American Academy of Pediatrics guidelines). The dotted vertical lines indicate term births (38-40 weeks gestation at delivery).

Supplementary Figure 5. Predicted probability of severe respiratory syncytial virus (RSV) lower respiratory tract infection (LRTI) requiring intensive care unit admission in the first year of life stratified by <29 or ≥29 weeks gestational age at delivery.


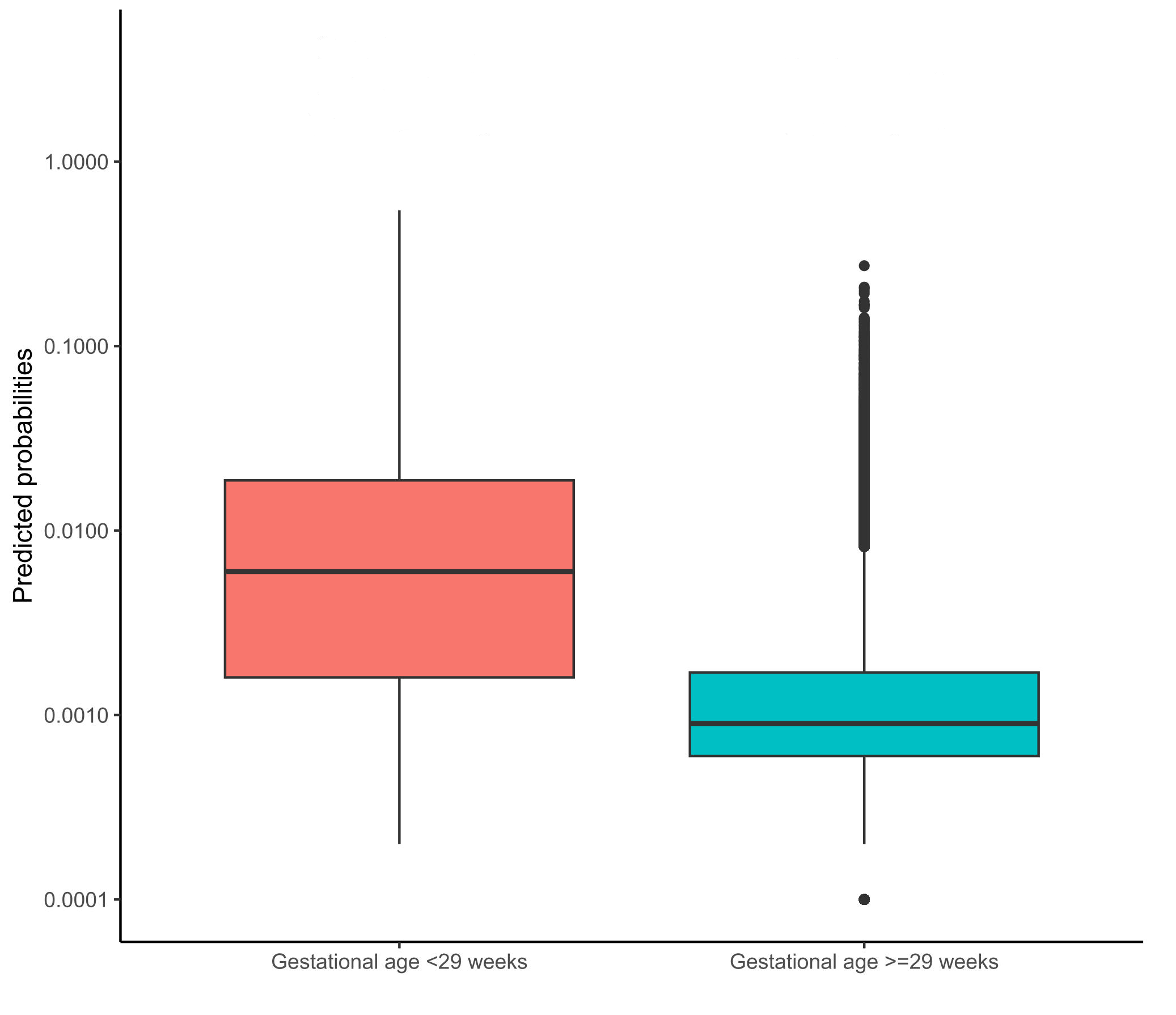


Predicted probabilities were estimated using univariate logistic regression.
